# The Specificity of Transgene Suppression in Plants by Exogenous dsRNA

**DOI:** 10.3390/plants11060715

**Published:** 2022-03-08

**Authors:** Konstantin V. Kiselev, Andrey R. Suprun, Olga A. Aleynova, Zlata V. Ogneva, Eduard Y. Kostetsky, Alexandra S. Dubrovina

**Affiliations:** 1 Laboratory of Biotechnology, Federal Scientific Center of the East Asia Terrestrial Biodiversity, Far Eastern Branch of the Russian Academy of Sciences, 690022 Vladivostok, Russia; kiselev@biosoil.ru (K.V.K.); suprun@biosoil.ru (A.R.S.); aleynova@biosoil.ru (O.A.A.); ogneva@biosoil.ru (Z.V.O.); 2 Department of Biochemistry, Microbiology and Biotechnology, Far Eastern Federal University, 690090 Vladivostok, Russia; kostetskiy.yeya@dvfu.ru

**Keywords:** exogenous dsRNA, plant foliar treatment, plant gene regulation, RNA interference, gene silencing, transgene

## Abstract

The phenomenon of RNA interference (RNAi) is widely used to develop new approaches for crop improvement and plant protection. Recent investigations show that it is possible to downregulate plant transgenes, as more prone sequences to silencing than endogenous genes, by exogenous application of double-stranded RNAs (dsRNAs) and small interfering RNAs (siRNAs). However, there are scarce data on the specificity of exogenous RNAs. In this study, we explored whether plant transgene suppression is sequence-specific to exogenous dsRNAs and whether similar effects can be caused by exogenous DNAs that are known to be perceived by plants and induce certain epigenetic and biochemical changes. We treated transgenic plants of *Arabidopsis thaliana* bearing the neomycin phosphotransferase II (*NPTII*) transgene with specific synthetic *NPTII*-dsRNAs and non-specific dsRNAs, encoding enhanced green fluorescent protein (*EGFP*), as well as with DNA molecules mimicking the applied RNAs. None of the *EGFP*-dsRNA doses resulted in a significant decrease in *NPTII* transgene expression in the *NPTII*-transgenic plants, while the specific *NPTII*-dsRNA significantly reduced *NPTII* expression in a dose-dependent manner. Long DNAs mimicking dsRNAs and short DNA oligonucleotides mimicking siRNAs did not exhibit a significant effect on *NPTII* transgene expression. Thus, exogenous *NPTII*-dsRNAs induced a sequence-specific and RNA-specific transgene-suppressing effect, supporting external application of dsRNAs as a promising strategy for plant gene regulation.

## 1. Introduction

Global population growth and the controversy surrounding transgenic plants require the development of new strategies and approaches to improve the nutritional properties of plants and their stress resistance. Numerous studies show the possibility of targeted down-regulation of plant gene expression by induction of RNA interference (RNAi) process [1,2,3,4]. RNAi is a conserved intracellular process of gene silencing that mediates plant resistance to undesirable nucleic acids and regulates expression of endogenous protein-coding genes [3]. The current RNAi-based crop protection tools are promising instruments used to purposefully reduce the expression of target genes and obtain desired plant phenotypes, such as improved pathogen resistance or various basic agronomic plant traits [1,4].

In the process of RNAi, long double-stranded RNA precursors (dsRNAs) are cleaved by specialized DICER-like (DCL) ribonucleases into small RNAs, such as small interfering RNA (siRNA) [5,6]. siRNAs are 5′-phosphorylated 20–24-nucleotide (nt)-long siRNA duplexes containing 2-nt 3′-overhangs at both ends. After processing, plant siRNAs are often modified by 2′-*O*-methylation at 3′ ends by HUA ENHANCER 1 (HEN1) to confer stability and prevent degradation by nucleases [5]. These small RNAs are then incorporated into the RNA-induced silencing complex (RISC), which cleaves homologous mRNAs or delays their translation [5,6]. The major RNAi-based process of plant protection and crop improvement involved the production of plants expressing dsRNAs or hairpin RNAs (hpRNAs), application of modified plant viruses for virus-induced gene silencing (VIGS), and the host-mediated silencing of pathogen genes for host-induced gene silencing (HIGS) [4,7,8]. However, the approaches require either the introduction of genetic modifications into the plant genome or the use of weakened plant viruses. Therefore, the development of new alternative approaches for gene regulation in plants and invading plant pathogens without genetic modifications (GM) is an important task in modern plant biotechnology.

In recent years, there has been an increasing number of studies showing that plants can uptake and process exogenous dsRNAs, hpRNAs, and siRNAs that were designed to complement important genes of plant pathogenic fungi or viruses [9,10]. These RNAs can be transferred to the fungi and induce RNAi processes, negatively affecting the fungal viability [11,12,13] or inducing plant virus resistance [14,15,16,17]. As a result, a new GM-free and eco-friendly strategy has emerged to protect plants from microbial infections, viral and insect attacks, and was termed as spray-induced gene silencing (SIGS) or ‘RNA vaccination’ [4,18]. This strategy is based on the exogenously induced RNAi (exoRNAi), which is induced by the application of the synthetic exogenous RNAs to the plant surfaces and is being developed as a promising alternative to transgenic plants and VIGS for plant pathogen protection. At the same time, there are few reports on the successful application of this strategy to downregulate expression of plant endogenous genes [19,20,21,22,23] or plant transgenes [24,25,26,27,28]. Several studies have reported downregulation of transgenes encoding green fluorescent protein (GFP), β-glucuronidase (GUS), yellow fluorescent protein (YFP), or neomycin phosphotransferase II (*NPTII*) during external application of dsRNA, siRNA, or hpRNA solutions of the leaf surface of tobacco or *Arabidopsis* [24,25,26,27,28]. Plant transgenes are known to be more prone for both local and systemic silencing in comparison with plant endogenes and endogene-resembling transgenes [29,30,31,32]. Therefore, application of exogenous RNAs for transgene silencing may be more easily achievable than targeting plant endogenes and would serve as a model system for triggering exoRNAi in plants.

Currently, there is a lack of studies analyzing the action of exogenous dsRNAs in terms of sequence- specificity of the transgene/endogene suppression effect. To date, there are no detailed studies where the effect of non-specific dsRNAs on plant target gene expression would be analyzed, i.e., it is necessary to clearly show whether different amounts of specific and non-specific dsRNAs have an effect on target gene expression. In addition, studies show that plants are able to perceive not only extracellular RNA [9,10,33,34], but also extracellular DNA (eDNA) molecules to induce certain signaling events in plant cells [35,36,37,38,39,40,41], For example, plants were shown to perceive eDNAs derived from plant pathogens [35,37], self-eDNA [39,40], and eDNA from other plant species [38,41], which are being considered to regulate plant innate immunity and self- and non-self recognition. Therefore, it is necessary to verify that the transgene suppressing effect is specific to dsRNA by analyzing the application of dsDNA molecules (similar in sequence and structure to the dsRNAs and siRNAs). Previously, we have shown that foliar application of synthetic dsRNAs and siRNAs designed to target the *NPTII* and *EGFP* transgenes in *A. thaliana* suppressed mRNA levels of these transgenes in the treated plants [26,27]. In this study, we aimed to evaluate whether exogenous transgene-encoding dsRNAs down-regulate the transgene targets specifically to their nucleotide sequence, and whether exogenous DNA molecules (identical in their sequence and modifications to the used exogenous RNAs) are capable of influencing transgene expression. Results of the present study showed that exogenous RNAs induce a sequence-specific and RNA-specific transgene-suppressing effect, indicating a significant potential of exogenous RNAs as a promising strategy in plant management.

## 2. Results

### 2.1. Exogenous dsRNAs Induce a Sequence-Specific Transgene Suppression in A. thaliana

**Figure 1:**
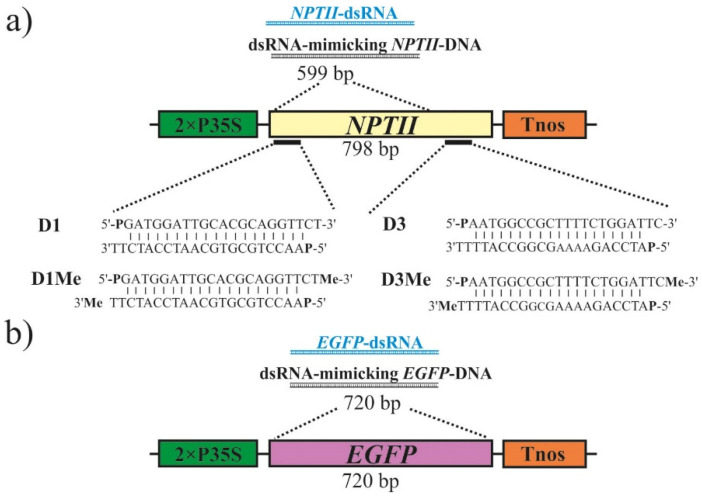
Schematic representation of the position and structures of the synthetic dsRNA, dsDNAs, and short DNA oligonucleotides used in this study for external plant treatments. The synthetic dsRNAs and DNAs were designed based on the pZP-RCS2 vector region encoding for the *NPTII* (**a**) or *EGFP* (**b**) transgenes. The long and short DNA molecules were designed to mimic dsRNA and siRNA, respectively. 2 × P35S—the doubled 35S promoter of the cauliflower mosaic virus (CaMV); *NPTII*—the neomycin phosphotransferase II (*NPTII*) gene; Tnos—nopaline synthase terminator. D1, D1Me, D3, D3Me—*NPTII*-encoding siRNA-mimicking DNA olignucleotides phosphorylated at 5′-ends and modified by 2′-*O*-methylation at 3′ ends.

**Figure 2:**
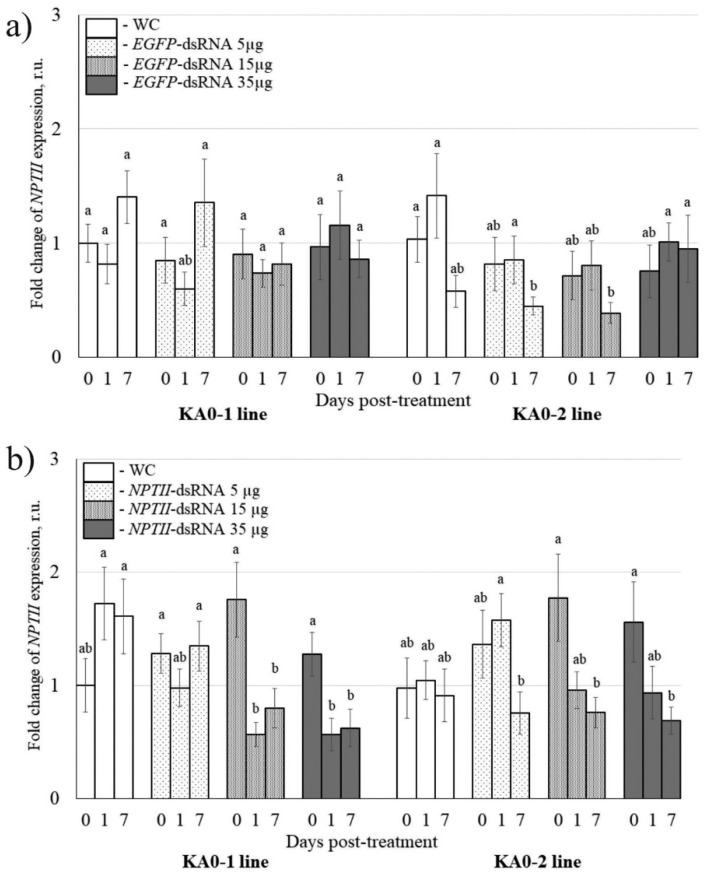
The analysis of *NPTII* mRNA levels in *Arabidopsis thaliana* in response to external application of synthetic *EGFP*-dsRNA and *NPTII*-dsRNA at different concentrations. (**a**) Quantification of *NPTII* mRNAs in *A. thaliana* in response to foliar application of *EGFP*-dsRNAs. (**b**) Quantification of *NPTII* mRNAs in *A. thaliana* in response to foliar application of *NPTII*-dsRNAs. WC—*A. thaliana* treated with sterile water (100 µL per plant). *EGFP*-dsRNA-5, 15, 35 and *NPTII*-dsRNA-5, 15, 35—the synthesized *EGFP*-dsRNA and *NPTII*-dsRNA were diluted in water to concentrations of 0.05, 0.15, and 0.35 µg/µL (100 µL per plant). KA0-1 and KA0-2—transgenic *Arabidopsis* lines bearing the *NPTII* transgene under the control of the doubled CaMV 35S promoter. The *NPTII* mRNAs were measured 1 day and 7 days post-treatment. qRT-PCR data are presented as mean ± SE (three independent experiments). Means on each figure followed by the same letter were not different using one-way analysis of variance (ANOVA), followed by the Tukey HSD multiple comparison test.

To verify that the transgene suppressing effect induced by exogenous dsRNAs is sequence-specific, we used two transgenic *Arabidopsis* lines KA0-1 and KA0-2, bearing the *NPTII* transgene under the control of the doubled cauliflower mosaic virus (CaMV) 35S promoter (Figure 1a). The KA0-1 and KA0-2 lines have previously been obtained and show high levels of *NPTII* transgene expression [27,42,43]. A large fragment of the *NPTII* gene (599 bp out of 798 bp) and the complete *EGFP* coding region (720 bp) was amplified by PCR for further in vitro transcription and dsRNA production (Figure 1a,b). We synthesized the *NPTII*-dsRNA (Figure 1a) and *EGFP*-dsRNA (Figure 1b) and treated the foliar surface of the two *NPTII*-transgenic *A. thaliana* lines to verify whether any observed effects on *NPTII* mRNA levels were specific to the sequence of the applied dsRNAs (Figure 2).

For the external application, 5, 15, or 35 µg of the synthesized dsRNAs was diluted in 100 µL of water and directly applied on the foliar surface of four-week-old *A. thaliana* (per individual rosette) by spreading with sterile individual brushes as described [26,27,38]. All leaves of one rosette were treated on both the adaxial and abaxial sides per each type of treatment in an independent experiment (Figure S1). In the present study, we treated four-week-old rosettes of *A. thaliana* at a late time of day (21:00–21:30) under low soil moisture conditions, since the appropriate plant age, late time of day, and low soil moisture at the time of dsRNA application were important parameters for successful plant transgene suppression according to our recent analysis [43]. Then, we compared *NPTII* mRNA levels in the *A. thaliana* treated either with *EGFP*-dsRNA (Figure 2a) or *NPTII*-dsRNA (Figure 2b). All three doses of the *EGFP*-dsRNA and the control water treatment did not considerably affect *NPTII* expression in the *NPTII*-transgenic plants one and seven days post-treatment (Figure 2a), while 15 and 35 µg of the *NPTII*-dsRNA considerably suppressed the *NPTII* mRNA levels (Figure 2b). For the KA0-1 line, a considerable *NPTII* transgene suppression effect was observed one and seven days after treatments with the *NPTII*-dsRNA, and, for the KA0-2 line, the detected effect was statistically significant seven days post-treatment.

To present an additional control of the *NPTII*-dsRNA specificity, we analyzed the effects of *NPTII*-dsRNA on the expression of four off-target *A. thaliana* genes, including *AtGAPDH*, *AtCHS*, *AtUBQ*, and *AtCML80*, which are not expressed from the 2xCaMV35S promoter (Figure S2). The analysis revealed that the exogenously applied *NPTII*-dsRNA did not affect expression of these genes at three different concentrations.

### 2.2. The Effect of Long dsDNAs on Transgene Expression

**Figure 3:**
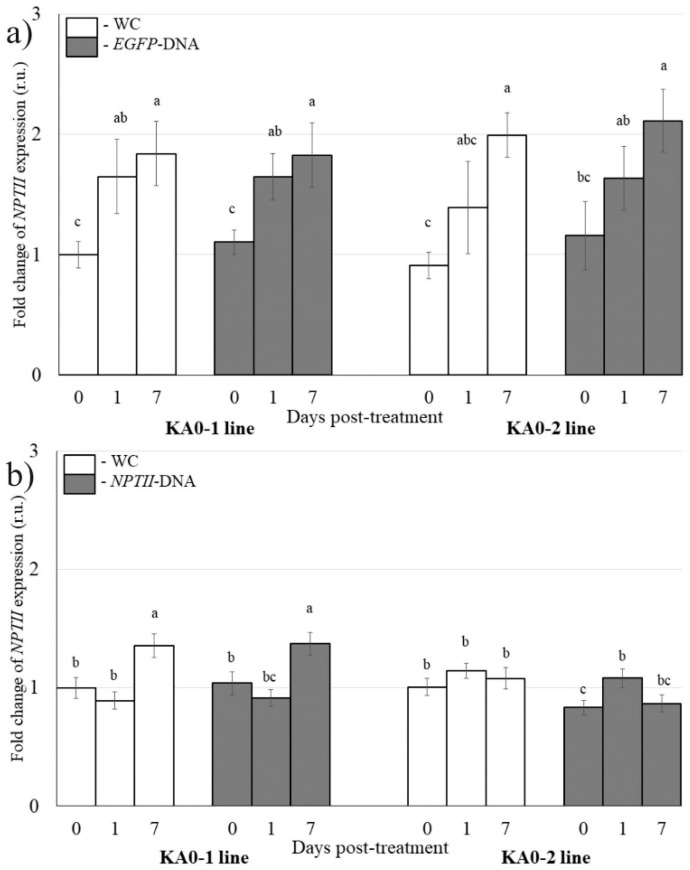
The analysis of *NPTII* mRNA levels in *Arabidopsis thaliana* in response to external application of synthetic *EGFP*-DNA (**a**) and *NPTII*-DNA (**b**) mimicking dsRNAs. The *NPTII* mRNAs were measured 1 day and 7 days post-treatment. WC—*A. thaliana* treated with sterile water (100 µL per plant); *EGFP*-DNA and *NPTII*-DNA—*A. thaliana* treated with synthetic DNAs. The synthesized *EGFP*- and *NPTII*-DNAs were diluted in water to a concentration of 0.35 µg/µL (100 µL per plant). KA0-1 and KA0-2—transgenic *Arabidopsis* lines bearing the *NPTII* transgene under the control of the doubled CaMV 35S promoter. qRT-PCR data are presented as mean ± SE (three independent experiments). Means on each figure followed by the same letter were not different using one-way analysis of variance (ANOVA), followed by the Tukey HSD multiple comparison test.

To verify whether exogenous application of double-stranded DNA (dsDNA) molecules mimicking dsRNAs can result in the suppression of a target gene, we designed and produced dsRNA-mimicking *NPTII*- and *EGFP*-dsDNAs (Figure 1a,b) identical to the large fragment of the *NPTII* gene (599 bp out of 798 bp), which was used for the synthesis the *NPTII*-dsRNAs. For external application, 35 µg of the synthesized dsDNA was diluted in 100 µL of water (per individual plant) and directly applied on the adaxial and abaxial leaf surface of four-week-old *A. thaliana* by spreading with sterile individual brushes as described above for *NPTII*-dsRNA. In contrast to *NPTII*-dsRNA, plant treatments with the complementary dsDNA did not have a substantial effect on the mRNA levels of the *NPTII* transgene (Figure 3a,b), while the applied dsDNA was detected on the leaves throughout the experiment (Figure S3).

### 2.3. The Effect of NPTII-DNA Oligonucleotides Mimicking siRNAs on Transgene Expression

**Figure 4:**
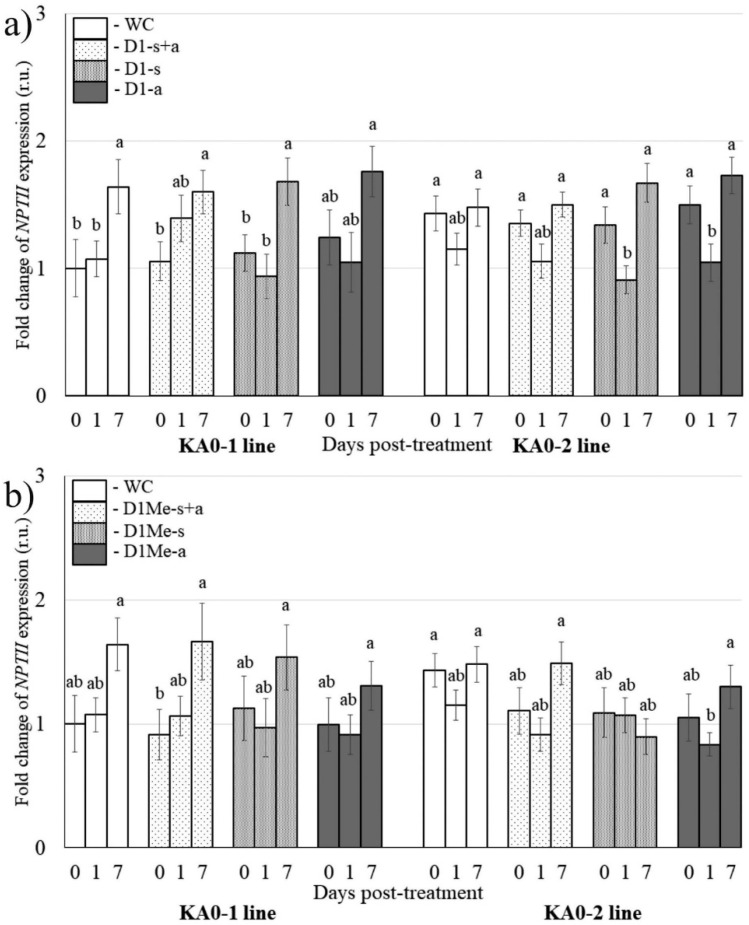
The analysis of *NPTII* mRNA levels in *Arabidopsis thaliana* in response to external application of synthetic *NPTII*-encoding D1 and D1Me DNA oligonucleotides designed to target the 5′ end of the transgene. (**a**) Foliar application of unmethylated single-stranded DNA oligonucleotides (D1-s, D1-a) and siRNA-mimicking DNA duplexes (D1-s+a). (**b**) Foliar application of methylated single-stranded DNA oligonucleotides (D1-s, D1-a) and siRNA-mimicking DNA duplexes (D1-s+a). WC—*A. thaliana* treated with sterile water (100 µL per plant); The *NPTII* mRNAs were measured 1 day and 7 days post-treatment. The DNA oligonucleotides were diluted in nuclease-free water to 50 pmol/μL (100 μL per plant). KA0-1 and KA0-2—transgenic *Arabidopsis* lines bearing the *NPTII* transgene under the control of the doubled CaMV 35S promoter. qRT-PCR data are presented as mean ± SE (three independent experiments). Means on each figure followed by the same letter were not different using one-way analysis of variance (ANOVA), followed by the Tukey HSD multiple comparison test.

**Figure 5:**
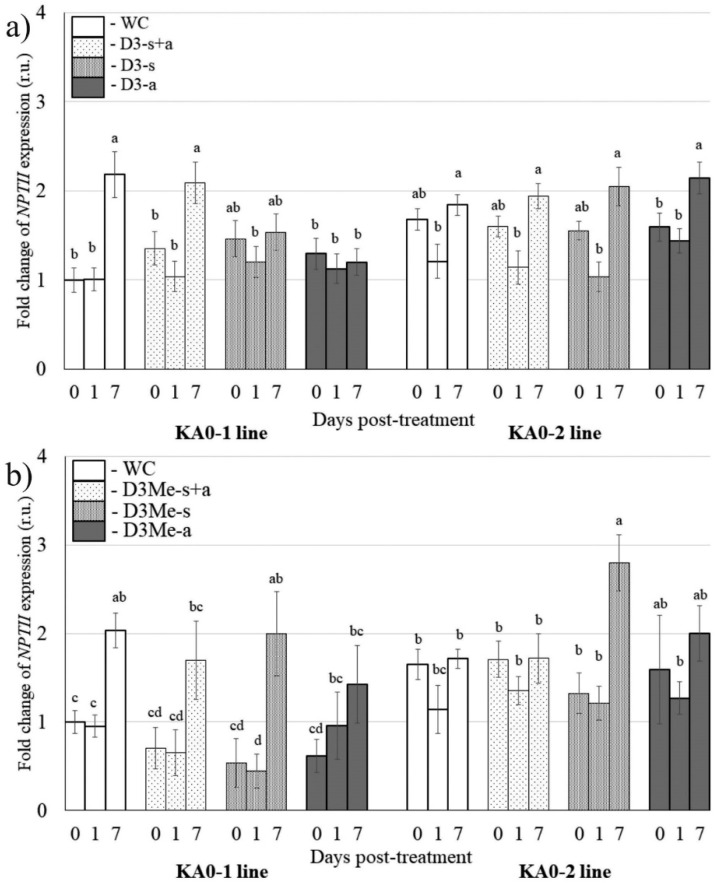
The analysis of *NPTII* mRNA levels in *Arabidopsis thaliana* in response to external application of synthetic *NPTII*-encoding D3 and D3Me DNA oligonucleotides designed to target the 3′ end of the transgene. (**a**) Foliar application of unmethylated single-stranded DNAs (D3-s, D3-a) and D3 siRNA-mimicking DNA duplexes (D3-s+a). (**b**) Foliar application of methylated single-stranded DNAs (D3Me-s, D3Me-a) and siRNA-mimicking DNA duplexes (D3Me-s+a). WC—*A. thaliana* treated with sterile water (100 µL per plant); The *NPTII* mRNAs were measured 1 day and 7 days post-treatment. The DNA oligonucleotides were diluted in nuclease-free water to 50 pmol/μL (100 μL per plant). KA0-1 and KA0-2—transgenic *Arabidopsis* lines bearing the *NPTII* transgene under the control of the doubled CaMV 35S promoter. qRT-PCR data are presented as mean ± SE (three independent experiments). Means on each figure followed by the same letter were not different using one-way analysis of variance (ANOVA), followed by the Tukey HSD multiple comparison test.

It has previously been shown that exogenous *NPTII*-siRNAs are capable of downregulating transgene expression in the *NPTII*-transgenic *Arabidopsis* [27]. To analyze the effect of DNA oligonucleotides mimicking the siRNAs on the *NPTII* transgene expression, four pairs of 21-nt long *NPTII*-encoding single-stranded DNA oligonucleotides targeting the 5′ end (D1 and D1-Me) and 3′ end (D3 and D3-Me) of the *NPTII* mRNAs were in vitro synthesized and HPLC purified (Figure 1a; Table S1). Then, we studied whether foliar application of all synthesized single-stranded DNA oligonucleotides (D1-s, D1-a, D3-s, D3-a, D1Me-s, D1Me-a, D3Me-s, D3Me-a) and siRNA-mimicking DNA duplexes (D1-s+a, D3-s+a, D1Me-s+a, and D3Me-s+a) at the concentration of 50 pmol/μL could alter the *NPTII* transgene transcript levels one and seven days post-treatment in two independent transgenic lines of *A. thaliana*, as compared with the plants before treatments (Figure 4 and Figure 5). All the complimentary DNA oligonucleotides contained a 5′ phosphate group and 2-nt 3′ overhangs at both ends when annealed. In addition, the D1-Me and D3-Me DNA oligonucleotides were modified at 3′ ends by 2′-*O*-methylation (Table S1; Figure 1a). The in vitro synthesized oligonucleotides were combined and annealed to form four siRNA-mimicking DNA duplexes—D1, D1Me, D3, and D3Me (Figure 1a). In our earlier study, 50 pmol/µL was chosen as the optimal concentration of the *NPTII*-siRNAs for plant foliar treatments due to the combination of effectiveness and lower cost of RNA oligonucleotide synthesis [27].

qRT-PCR revealed that foliar application of both the single-stranded D1 and D3 DNA oligonucleotides (D1-s, D1-a, D3-s, D3-a) and the DNA duplexes (D1-s+a, D3-s+a) to the foliar surface of *A. thaliana* did not considerably reduce mRNA levels of the *NPTII* transgene in the plant one and seven days after treatments (Figure 4a and Figure 5a). Similarly, application of both single-stranded and annealed D1Me and D3Me DNA oligonucleotides did not show a considerable and stable *NPTII*-downregulation effect (Figure 4b and Figure 5b). We noticed a considerable increase in *NPTII* expression seven days after application of both water and the DNA oligonucleotides in the KA0-1 line (Figure 4 and Figure 5). This increase can be explained by the cumulative effect of the transgene mRNA accumulation over time, which was more pronounced for this particular plant line.

## 3. Discussion

Currently, RNAi-based technologies are recognized as a promising and safe approach for plant protection against fungal pathogens, viruses, and insects [10,44,45]. Recent studies increasingly demonstrate the ability of plants to uptake and process exogenous RNAs and the effectiveness of exogenous RNAs against plant pathogens and pests [9,44,46]. Recent studies reported on the substantial induction of plant viral [14,15,16,17], fungal [11,12,13], and insect [47,48] resistance after external application of dsRNAs, hpRNAs, and siRNAs targeting essential genes of the pathogens. The exogenously applied dsRNAs entered not only fungal cells but also plant cells and plant vascular system. They were processed to siRNAs and initiated RNA silencing, supporting the conclusion that observed resistance is an RNAi-mediated process [11,16]. The exogenous dsRNAs and siRNAs have been shown to spread systemically from the treated leaves to non-treated ones [14,15]. Currently, exogenous RNAs are considered as a new class of safe pesticides, insecticides, or antiviral agents [10,44,49].

Several studies show that it is possible to down-regulate plant transgenes, including *GFP*, *GUS*, and *YFP* [24,25,26,27,28] or plant endogenous genes, including R2R3-MYB transcription factors, sugar transporter gene, a downy mildew susceptibility gene, and a chalcone synthase gene [19,20,21,22,23]. However, the number of reports on the effect of exogenous RNAs on the expression of specific plant gene targets is small and there are scarce data on the specificity and effectiveness of this approach. Plant transgenes are usually under the control of strong promoters, ensuring a high level of constitutive expression and, thus, elevating possible production of aberrant mRNAs, e.g., truncated and/or read-through transcripts, leading to the amplification of transgene silencing [32,42]. Current studies show that plant transgenes are more sensitive to RNAi-mediated silencing than endogenes due to the absence of introns and other untranslated regions, as well as to a high level of aberrant mRNAs that turn into secondary small RNAs [29,30,31,32]. In a recent study, we treated *NPTII-* and *EGFP*-transgenic adult plants of *A. thaliana* with long *NPTII*- and *EGFP*-encoding dsRNAs, which led to a considerable suppression of both transgenes in the treated leaves [26]. Then, we have shown that foliar application of *NPTII*-siRNAs to *NPTII*-transgenic *Arabidopsis* down-regulated *NPTII* mRNA transcript levels, with a higher and more consistent effect being observed for siRNAs methylated at 3′ ends [27]. Surprisingly, the previous dsRNA-based study [26] showed that the *NPTII* mRNA level was suppressed in *A. thaliana* treated with the *EGFP*-dsRNA, and vice versa. On one side, we detected the read-through transcripts that included both *NPTII* and *EGFP* in the transgenic plants and the *EGFP*-derived siRNA after application of *NPTII*-dsRNA, which suggested that *NPTII* silencing was accompanied by the phenomenon of transitivity [26]. On the other side, it is necessary to verify that the exogenously applied dsRNAs did not induce unspecific transgene suppression. Therefore, in this study, we used *A. thaliana* plants transformed with a single *NPTII* transgene for foliar treatments with both *NPTII*- and *EGFP*-dsRNAs and showed that none of the *EGFP*-dsRNA doses resulted in a significant decrease in *NPTII* expression in the *NPTII*-transgenic *A. thaliana*, while the specific *NPTII*-dsRNA significantly reduced *NPTII* expression. This result indicates the sequence specificity of the observed transgene suppression effect in relation to the nucleotide sequences of the applied exogenous dsRNA.

Current studies show that plants are able to perceive not only extracellular RNA [9,10,33,34], but also extracellular DNA (eDNA) molecules [35,36] that are able to induce certain signaling events in plant cells and enhance the immune response of plants. Plants were shown to perceive eDNAs derived from plant pathogens [35,37], self-eDNA [39,40], and eDNA from other plant species [38,41], which are being considered to regulate plant innate immunity and self- and non-self recognition. For example, Paungfoo-Lonhienne et al. [36] have shown that labeled external phosphorothioate DNA oligonucleotides were taken up by plant root hairs and pollen, used as a phosphorus source, and stimulated root and pollen tube growth. It is known that heterologous DNA and RNA, which are released from plant tissue damage, act as damage-associated molecular patterns (DAMPs), while microbial DNA and RNA act as microbe-associated molecular pattern (MAMPs) or pathogen-associated molecular pattern (PAMP) [50]. The results of Yakushiji et al. [35] suggested that non-methylated bacterial CpG DNA, as a MAMP, induced defense responses in *Arabidopsis* and that non-methylated DNA seems to be translocated into the cytoplasm by endocytosis.

Other studies indicated that plant self eDNA has a role as a DAMP in common bean [51] and lettuce [38] depending on phylogenetic closeness and inducing epigenetic, genetic and biochemical changes within the plant. Furthermore, the data show that plant cells distinguish self- from non-self eDNA [39,40,52]. The available studies have shown that eDNA affects ROS production, DNA methylation, and induces defense-related responses, such as altered gene expression associated with oxidative burst and production of secondary metabolites within plants [38,51,52]. However, the induced specific signaling pathways and plant responses after eDNA perception remain largely unknown. External dsRNA is also known to be implicated in plant immunity and represent a genuine pathogen-associated molecular pattern (PAMP) [26,27]. Since dsRNA is a key component of RNAi and is implicated in plant immunity mechanisms, one can propose that eDNA molecules could also be involved in this process. Therefore, we believe that it was important to verify whether the exogenously induced transgene silencing effects may be attributed not only to RNA, but also to DNA molecules. In contrast to external dsRNA and siRNA applications presented in this study and previous studies [26,27], plant treatments with the complementary dsDNAs and short DNA oligos did not have a substantial effect on the mRNA levels of the *NPTII* transgene. The results obtained highlighted the uniqueness of dsRNA and siRNA molecules in their ability to induce silencing of target transgene sequences in the plant genome.

In conclusion, external application of unspecific dsRNA, transgene-specific DNA oligonucleotides and long DNA molecules did not induce transgene silencing in *A. thaliana*. Thus, exogenous dsRNAs trigger a sequence-specific and RNA-specific suppression of plant transgenes, supporting exogenous dsRNA and siRNA application as a promising strategy for plant gene regulation.

## 4. Materials and Methods

### 4.1. Plant Material and Growth Conditions

*Arabidopsis thaliana* (cv. Columbia) plants were grown in pots filled with commercially available rich soil “Universal Soil” (Fasko, Moscow, Russia) in a controlled environmental chamber at 22 °C (Sanyo MLR-352, Panasonic, Japan) on a 16-h light/8-h dark cycle (light intensity ~120 μmol·m^−2^·s^−1^). The soil consisted of riding peat, lowland peat, sand, limestone (dolomite) flour, complex mineral fertilizer with microelements. The content of nutrients available to plants (mg/kg) was not less than: Nitrogen—350; Phosphorous—400; Potassium—500; pH—6–7. To generate the *NPTII*-overexpressing plants, we used the binary plasmid construct pZP-RCS2-*NPTII*, which was kindly provided by Professor Alexander Krichevsky (State University of New York, Stony Brook, USA) [53]. This construct carries the *NPTII* gene under the control of the doubled CaMV 35S promoter. *A. thaliana* was transformed previously by the floral-dip method as described [27,54]. The two transgenic lines KA0-1 and KA0-2 selected for use in this study were T_4_ homozygous plants with a single copy insertion obtained previously [27]. The seeds of *A. thaliana* were sterilized and plated as described [27]. One-week-old *A. thaliana* seedlings were planted to pots (7 × 7 cm) containing 100 g of commercially available rich soil and were grown under the conditions described above.

### 4.2. dsRNA Production

dsRNAs of *NPTII* and *EGFP* were synthesized using the T7 RiboMAX™ Express RNAi System (Promega, Madison, WI, USA). The cloned large fragment of *NPTII* (GenBank AJ414108, 599 bp out of 798 bp) and complete *EGFP* (GenBank U55762/AY818363, 720 bp) were amplified by PCR using pZP-RCS2-*EGFP*-*NPTII* plasmid [53] for the following in vitro transcription and dsRNA production. The T7 promoter sequence was introduced into both the 5′ and 3′ ends of *EGFP* and *NPTII* in a single PCR for each gene using primers listed in Table S2. The PCRs were performed in the Bis-M111-02-48 Thermal Cycler (Novosibirsk, Russia) programmed according to T7 RiboMAX™ Express RNAi System instructions. PCR was carried out in a final volume of 30 µL, containing 1× Taq reaction buffer with 3 mM MgCl_2_, 0.5 µL plasmid DNA (50 ng), 200 µM dNTPs, 0.2 µM of each primer, and 2.0 units of Taq DNA polymerase (Evrogen, Moscow, Russia). After an initial denaturation at 95 °C for 5 min, the first 5 cycles were performed as follows: 95 °C for 10 s, 65 °C (*NPTII*) or 66 °C (*EGFP*) for 10 s, 72 °C for 38 s (*NPTII*) or 45 s (*EGFP*), followed by 35 cycles each of 95 °C for 10 s and 72 °C for 48 s (*NPTII*) or 55 s (*EGFP*). After a final extension at 72 °C for 5 min, PCR fragments were loaded on 1% agarose gel and purified by the Cleanup Standard kit (Evrogen).

The obtained PCR products were used as templates (0.2 µg per probe) for in vitro transcription and dsRNA synthesis following the manufacturer’s protocol. The resultant dsRNAs were analyzed by gel electrophoresis and spectrophotometry to estimate dsRNA purity, integrity, and amount.

### 4.3. dsRNA Application

The dsRNAs were applied to all leaves of the individual four-week-old rosettes of wild-type *A. thaliana* by spreading with individual soft brushes (natural pony hair) sterilized by autoclaving [26,43] (Figure S1; Video S1). For each dsRNA treatment, 35 µg of the dsRNA were diluted in 100 µL of nuclease-free water and applied to the foliar surface: all leaves of one rosette were treated on both the adaxial (upper) and abaxial (lower) sides for each type of treatment (three independent experiments with one individual plant per each treatment in an independent experiment). One plant of *A. thaliana* was treated with the dsRNA of each type (100 µL) and one plant—with sterile filtered water (100 µL) in each independent experiment. The dsRNAs in all experiments were applied to four-week-old rosettes of *A. thaliana* at a late time of day (21:00–21:30) under low soil moisture conditions, since an appropriate plant age, late time of day, and low soil moisture (at the time of dsRNA application) were important parameters for successful *NPTII* suppression according to our recent analysis [43]. Soil water content before dsRNA treatments was 50–60%.

### 4.4. Production and Application of Long NPTII-DNAs and Short DNA Oligonucleotides

Four pairs of 21-nt long single-stranded DNA oligonucleotides designed to target the 5′ end (D1 and D1-Me) and 3′ end (D3 and D3-Me) of the *NPTII* mRNAs were in vitro synthesized, modified, and HPLC purified by Syntol (Moscow, Russia). All the in vitro synthesized complimentary DNA oligonucleotides contained a 5′ phosphate group and 2-nt 3′ overhangs at both ends when annealed. In addition, the D1-Me and D3-Me oligonucleotides were modified at 3′ ends by 2′-*O*-methylation. The DNA oligonucleotide sequences are presented in Table S1 and Figure 1a.

To obtain siRNA-mimicking DNA duplexes, equal volumes of the single-stranded oligonucleotides diluted to a concentration of 100 pmol/µL were combined and annealed at 90 °C for 1 min. The mixture was slowly cooled to room temperature. The final concentration of annealed oligonucleotides was 50 pmol/µL. 100 µL of each single-stranded DNA oligonucleotide, siRNA-mimicking DNA duplex, or 100 µL of nuclease-free water were applied onto the leaf surface of *A. thaliana* by spreading with individual soft brushes as described above for dsRNAs. Then, dsRNA-mimicking dsDNA encoding the same fragment of the *NPTII* gene (599 bp out of 798 bp) was synthesized by PCR using the pZP-RCS2-*EGFP*-*NPTII* plasmid [53] as a template. The PCR was carried out in a final volume of 30 µL as described above for *NPTII*-dsRNA production. To guarantee a sufficient amount of the DNA, the reaction was performed in 50 replicates. 35 µg of the dsDNA were diluted in 100 µL of nuclease-free water and applied onto the leaf surface of four-week-old *A. thaliana* by spreading with individual soft brushes as described above for dsRNAs.

### 4.5. RNA and DNA Isolation and REVERSE Transcription

For RNA isolations, we used fifth to eight large adult leaves of *A. thaliana* [42]. A typical adult leaf of *A. thaliana* was collected from the same individual plant at three time points for each type of treatment (before treatment, one day, and seven days post-treatment) in an independent experiment. One typical adult leaf was used for the nucleic acid isolation each time from the same plant. Total RNA was isolated using the cetyltrimethylammonium bromide (CTAB)-based protocol [55] and complementary DNAs were synthesized as described [56]. DNA was isolated as described earlier [57].

### 4.6. Gene Expression Analysis by Quantitative RT-PCR

The reverse transcription products were amplified by PCR and verified on the absence for DNA contamination using primers listed in Table S2. The qRT-PCRs were performed with SYBR Green I Real-time PCR dye and a real-time PCR kit (Evrogen, Moscow, Russia) as described [26] using *GAPDH* and *UBQ* as two internal controls selected in previous studies as relevant reference genes for qRT-PCRs on *Arabidopsis* [58]. The expression was calculated by the 2^−ΔΔCT^ method [59]. qRT-PCR data shown were obtained from three independent experiments and are averages of six technical replicates for each independent experiment (three qPCR reactions normalized to *GAPDH* and three qPCR reactions normalized to *UBQ* expression). A no-template control was included in every assay.

All gene identification numbers and primers used in PCR are listed in Table S2.

### 4.7. Statistical Analysis

The data are presented as mean ± standard error (SE) and were evaluated by one-way analysis of variance (ANOVA), followed by the Tukey HSD multiple comparison test performed in Excel using the XLSTAT software. A value of *p* < 0.05 was considered significant. Three independent experiments were performed for each type of analysis. In each independent experiment (Figure S1), one individual plant was taken from which one leaf was cut off at each point for each type of treatment.

## Data Availability

Not applicable.

## Supplementary Materials

The following are available online at https://www.mdpi.com/article/10.3390/plants11060715/s1, Video S1: dsRNA spreading with sterile individual soft brushes. Table S1: In vitro-synthesized single-stranded DNA olignucleotides. Table S2: Primers used in RT-PCR and qRT-PCRs. Figure S1: Schematic representation of the experiments conducted on 4-week-old *Arabidopsis thaliana* for the analysis of *NPTII* mRNA levels after foliar dsRNA and dsDNA treatments. Figure S2: Expression of the *AtGAPDH*, *AtCHS*, *AtUBQ*, and *AtCML80* genes in four-week-old *Arabidopsis thaliana* in response to external application of synthetic *NPTII*-dsRNA at different concentrations. Figure S3: The analysis of *NPTII* DNA levels using primers designed to align inside the *NPTII* transgene fragments, which have been used for synthesis of the corresponding dsDNA in four-week-old *Arabidopsis thaliana*.
