# Supplementary material for: The Specificity of Transgene Suppression in Plants by Exogenous dsRNA
Source: Plants (Basel). 2022 Mar 8;11(6):715. doi: 10.3390/plants11060715 (PMC8954795; doi:10.3390/plants11060715)

Table S1 In vitro-synthesized single-stranded DNA olignucleotides.

		5' - 3' RNA oligonucleotides	Modification
1	D1-s	5'-GATGGATTGCACGCAGGTTCT	5′ phosphate
2	D1-a	5'-AACCTGCGTGCAATCCATCTT	
3	D1Me-s	5'-GATGGATTGCACGCAGGTTCT	5′ phosphate
4	D1Me-a	5'-AACCTGCGTGCAATCCATCTT	2′-*O*-methyl at 3′ end
5	D3-s	5'-AATGGCCGCTTTTCTGGATTC	5′ phosphate
6	D3-a	5'-ATCCAGAAAAGCGGCCATTTT	
7	D3Me-s	5'-AATGGCCGCTTTTCTGGATTC	5′ phosphate
8	D3Me-a	5'-ATCCAGAAAAGCGGCCATTTT	2′-*O*-methyl at 3′ end

Table S2 Primers used in RT-PCR and qRT-PCRs.

Gene name (GenBank acc. no)	Primer name	Primers, 5′-3′
Primers for cDNA check-up on DNA contamination, 5′-3′		
AtGAPDH (NM_111283)	AtGapdh-s<br>AtGapdh-a	5′CTG GAA TGT CTT TCC GTG TC<br>5′ATT CGT TGT CGT ACC ATG AC
Primers for PCR and real-time PCR, 5′-3′		
NPTII (AJ414108)	nptII-realS<br>nptII-realA	5′TTGCTGAAGAGCTTGGCGGCGAAT<br>5′TCAGAAGAACTCGTCAAGAAGG
AtGAPDH (NM_111283)	AtGapdh-real-s<br>AtGapdh-real-a	5′TTG GTG ACA ACA GGT CAA GCA<br>5′AAA CTT GTC GCT CAA TGC AAT
AtUBQ (NM_001084884)	AtUBQ-realS<br>AtUBQ-realA	5′GGCCTTGTATAATCCCTGATGAATAAG<br>5′AAAGAGATAACAGGAACGGAAACATAGT
Specific primers for dsRNA design, 5′-3′		
NPTII (AJ414108)	npt-T71-s<br>npt-T72-a	5′TAATACGACTCACTATAGGGAGAATGTGGATTGAACAAG ATGGATTG<br>5′TAATACGACTCACTATAGGGAGATCCACCATGATATTCG GCAAGCAG

Figure S1. Schematic representation of the experiments conducted on 4-week-old *Arabidopsis thaliana* for the analysis of *NPTII* mRNA levels after foliar dsRNA and dsDNA treatments. (a) Total RNA isolation from *A. thaliana* before treatments; (b) Foliar application of water (upper panel) or nucleic acids (lower panel) by spreading with sterile individual brushes; (c) Total RNA isolation from *A. thaliana* 1 day post-treatment; (d) Total RNA isolation from *A. thaliana* 7 days post-treatment. For RNA isolations, a typical adult leaf of *A. thaliana* was collected from the same individual plant at three time points for each type of treatment in an independent experiment. Red crosses depict leaves used for RNA isolation before treatment, white cross – 1 day post-treatment, and blue cross – 7 days post-treatments. All leaves of plant 1 (upper panel) were treated with water or with dsRNA or dsDNA on both the adaxial and abaxial sides. All leaves of plant 2 (lower panel) were treated with dsRNA or dsDNA on both the adaxial and abaxial sides.

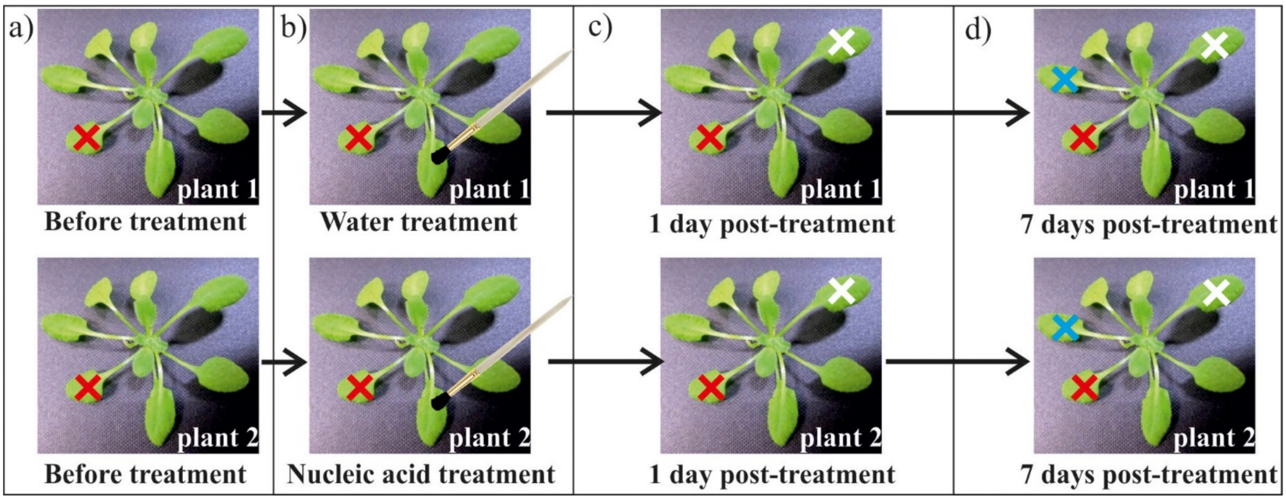

Figure S2. Expression of the *AtGAPDH* (a), *AtCHS* (b), *AtUBQ* (c), and *AtCML80* (d) genes in four-week-old *Arabidopsis thaliana* in response to external application of synthetic *NPTII*-dsRNA at different concentrations. WC – *A. thaliana* treated with sterile water (100 μL per plant). *EGFP*-dsRNA-5, 15, 35 and *NPTII*-dsRNA-5, 15, 35 — the synthesized *EGFP*-dsRNA and *NPTII*-dsRNA were diluted in water to concentrations of 0.05, 0.15, and 0.35 μg/μL (100 μL per plant). KA0-1 and KA0-2 – transgenic *Arabidopsis* lines bearing the *NPTII* transgene under the control of the doubled CaMV 35S promoter. The *AtGAPDH*, *AtCHS*, *AtUBQ*, and *AtCML80* mRNAs were measured 1 day and 7 days post-treatment. qRT-PCR data are presented as mean ± SE (three independent experiments). Means on each figure followed by the same letter were not different using one-way analysis of variance (ANOVA), followed by the Tukey HSD multiple comparison test.

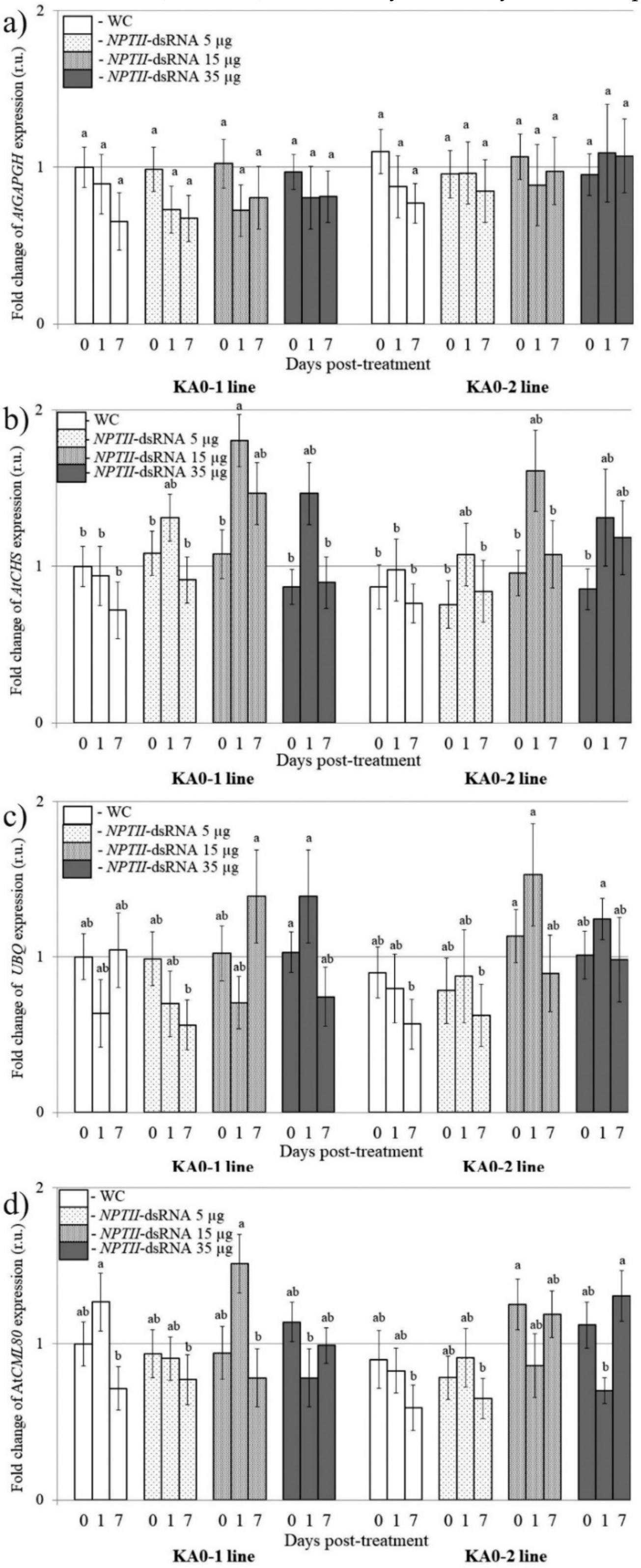

Figure S3. The analysis of *NPTII* DNA levels using primers designed to align inside the *NPTII* transgene fragments, which have been used for synthesis of the corresponding dsDNA in four-week-old *Arabidopsis thaliana*. The analysis has been performed to analyze dsDNA stability 1 and 7 days post treatment. WC — *A. thaliana* treated with sterile water (100 μL per plant); *NPTII*-DNA — *A. thaliana* treated with synthetic dsDNAs. The synthesized *NPTII*-DNAs were diluted in water to a concentration of 0.35 μg/μL (100 μL per plant). KA0-1 and KA0-2 – transgenic *Arabidopsis* lines bearing the *NPTII* transgene under the control of the doubled CaMV 35S promoter. qRT-PCR data are presented as mean ± SE (three independent experiments). Means on each figure followed by the same letter were not different using one-way analysis of variance (ANOVA), followed by the Tukey HSD multiple comparison test.

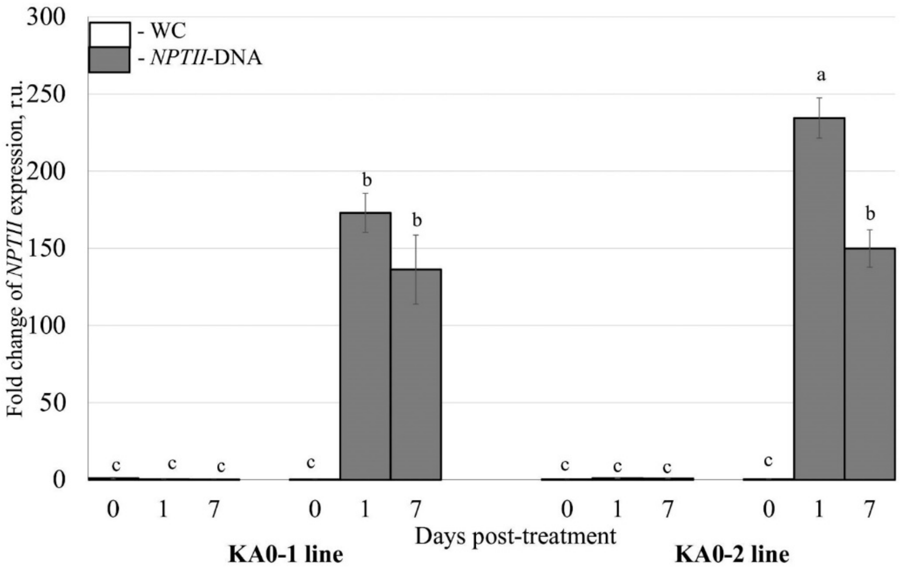

Supplement: Supplementary file 1 [file plants-11-00715-s001.zip › Supplementary-10.pdf]
